# Research on Micro/Nano Surface Flatness Evaluation Method Based on Improved Particle Swarm Optimization Algorithm

**DOI:** 10.3389/fbioe.2021.775455

**Published:** 2021-12-15

**Authors:** Han Shu, Chunlong Zou, Jianyu Chen, Shenghuai Wang

**Affiliations:** School of Mechanical Engineering, Hubei University of Automotive Technology, Shiyan Hubei, China

**Keywords:** flatness error, micro/nano surface, improved particle swarm optimization algorithm, minimum zone method, uncertainty, AFM

## Abstract

Flatness error is an important factor for effective evaluation of surface quality. The existing flatness error evaluation methods mainly evaluate the flatness error of a small number of data points on the micro scale surface measured by CMM, which cannot complete the flatness error evaluation of three-dimensional point cloud data on the micro/nano surface. To meet the needs of nano scale micro/nano surface flatness error evaluation, a minimum zone method on the basis of improved particle swarm optimization (PSO) algorithm is proposed. This method combines the principle of minimum zone method and hierarchical clustering method, improves the standard PSO algorithm, and can evaluate the flatness error of nano scale micro/nano surface image data point cloud scanned by atomic force microscope. The influence of the area size of micro/nano surface topography data on the flatness error evaluation results is analyzed. The flatness evaluation results and measurement uncertainty of minimum region method, standard least squares method, and standard PSO algorithm on the basis of the improved PSO algorithm are compared. Experiments show that the algorithm can stably evaluate the flatness error of micro/nano surface topography point cloud data, and the evaluation result of flatness error is more reliable and accurate than standard least squares method and standard PSO algorithm.

## Introduction

A typical engineering surface consists of a range of spatial frequencies (Raja et al., 2002). The high-frequency or short-wavelength components are referred to as roughness, the medium frequencies as waviness, and low frequency components as form. Flatness error is one of the important standards to evaluate the quality of micro/nano surface. The surface flatness error of calibration samples, semiconductors, wafers, and other micro/nano devices is required to be extremely high. For example, extreme ultraviolet lithography wafers (Nutsch et al., 2008) require the highest flatness error of the exposure surfaces because the depth of focus is affected. Because of flatness defects, the exposure process suffers from defocusing. Those topography defects are expected to get a severe yield limiting factor of future technology generations. At present, the step height standard (SHS) samples are commonly used to calibrate scanning electron microscopy, scanning tunneling microscopy, and atomic force microscopy (AFM). It is a kind of micro/nano device with ultra-high precision nominal height. It is an important medium to transfer the micro/nano size of the standard device of national metrology standard department to the actual production and manufacturing (Yang et al., 2018). Therefore, its surface quality requirements are very high, and its flatness error index will directly affect the subsequent step height evaluation results. Therefore, it is of great practical significance to evaluate the flatness error efficiently and accurately.

**FIGURE 1:**
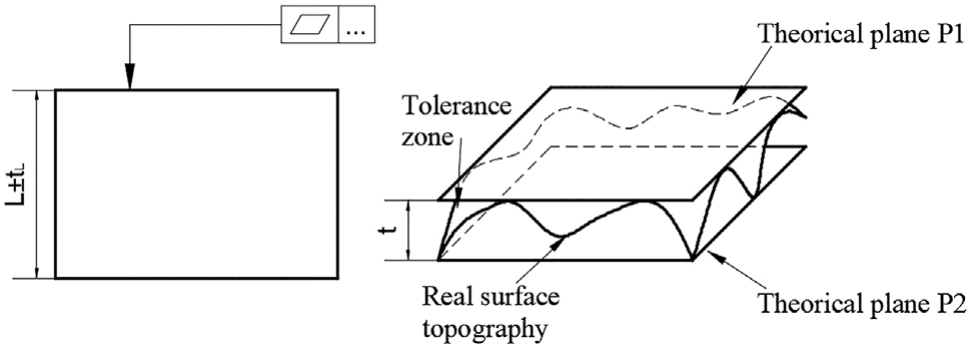
Flatness error deviation.

The methods of evaluating flatness error include minimum zone method, least squares method, diagonal plane method, and three-far-point plane method. Among them, the minimum zone method and the least squares method meet the ISO standard. The ISO 6318 provides two criteria to evaluate the flatness error: the least squares criterion (LSC) (Moulai-Khatir et al., 2018) and the minimum zone criterion (Jalid et al., 2015). The LSC is widely used in the industry for assessing the dimensions and geometric tolerances. It is simple to compute, easy to implement in computer processing, and provides unique solutions. The minimum zone method is introduced in the ISO 1101 (ISO, 2017) standard. Figure 1 shows that flatness error is defined as the minimum distance between two parallel planes P1 and P2 containing all data points. However, the standard does not define the way of obtaining the minimum zone solution. In the existing literature, most of these algorithms are only used to evaluate the flatness error of the measurement results of micro scale CMM equipment. Whether it can be used for micro/nano surface topography is unknown. In addition, flatness error values obtained using different methodologies are not uniform as it is in the case of least squares method. For now, the success of the applied the minimum zone methodology is reflected on the approximation of the assessed flatness error to the exact value. Štrbac et al. (2020) proposed a large number of factors that affect measurement accuracy and give comparison between the efficacies of different the minimum zone methodologies. However, it can only be made for the same set of sampled points in coordinate metrology. Cui et al. (2008) applied particle swarm optimization (PSO) to the optimization calculation of flatness error, which can well to solve the optimization problems with nonlinear optimization objective function or multi-parameters. Moreover, the calculation accuracy of PSO is better than that of the least squares method, which is equivalent to that of other methods that meet the minimum zone conditions defined in the standard. It can obtain high-precision results and is simple and easy to implement. However, this method is easily affected by equipment noise and surface defects in the process of micro/nano surface topography treatment, resulting in unsatisfactory evaluation results.

In this paper, the flatness error evaluation of micro nano surface topography is studied. There are five contributions of this paper.

1) The existing bioinspired algorithms and traditional algorithms for processing two-dimensional surface topography data and three-dimensional surface topography data are reviewed, and their advantages and disadvantages are analyzed.
2) The characteristics of micro and nano surface topography data measured by atomic force microscope were analyzed.
3) Inspired by the minimum region method, hierarchical clustering algorithm and PSO algorithm, a minimum region method on the basis of the improved PSO algorithm is proposed to evaluate the flatness of micro and nano surface topography data.
4) The performance of the algorithm is analyzed by measuring the surface topography data of the SHS samples and compared with other classical algorithms.
5) The effects of different surface topography zone size on the results were analyzed.

The remaining chapters of this paper are arranged as follows: The related work is discussed in *Related Work*, and then, the step surface topography data are preprocessed in *Data Preprocessing*. In *Flatness Error Evaluation Method*, the principle of minimum region method on the basis of the improved PSO algorithm is studied. *Experimental Results and Analysis* gives the experimental results and analysis, and *Conclusion* summarizes the summary of this paper and the future research direction.

## Related Work

For the evaluation of micro/nano surface parameters, the evaluation methods are mainly selected according to the complexity of the corresponding micro/nano surface. For the surface with complex surface shape, we first use the segmentation method to separate the parts that we are interested in and then use the relevant evaluation methods to evaluate. The surface with simple structure and obvious characteristics can be evaluated according to the surface data type.

The micro/nano surface topography data are roughly divided into two-dimensional image data and three-dimensional image data. For the two-dimensional image data, Blunt and Xiao (2011) used the morphological segmentation method to segment the complex micro/nano surface topography features in the hard disk laser area. It provides the possibility to effectively describe the geometry of micro/nano surfaces. Pin et al. (2016) completed the segmentation of features in diamond grinding surface topography by marker-based watershed segmentation method. Among them, to get better segmentation effect, he tested different diamond disks and found that the segmentation results will decrease with the increase of diamond disk radius. Macaulay et al. (2014) conducted comparative experiments on the surface topography of laser textured silicon nitrides disk by height threshold method, gradient threshold method, morphological segmentation, and active contour method. It is concluded that the active contour method and morphological segmentation have good robustness and stable output results. Height threshold method and gradient threshold method have better sensitivity and more flexible parameter setting. For 3D image data, Lou et al. (2020) used the watershed segmentation method to segment the 3D features of triangular mesh comet surface, and extended Maxwell theory to the 3D watershed segmentation of triangular mesh surface. Jiang et al. (2021) proposed a multi-task semantic segmentation model, which not only can obtain the location information of the object but also further obtain the semantic information of the object.

When we get the data interested in surface topography, we can use some corresponding evaluation methods for evaluation. The flatness error evaluation mainly adopts the least squares method and the minimum zone method. There are many ways to determine the minimum zone. Many scholars are committed to this research. Xu et al. (2018) proposed a region search algorithm to improve the effectiveness and practicability of the flatness error evaluation method. The method determines the initial datum plane according to the existing measurement points, rotates the coordinate system to the *x-y* plane parallel to the initial datum plane, and then gradually determines the new coordinate A and B values through orthogonal test to determine the error size. Arezki et al. (2018) studied and implemented two minimum zone fitting algorithms, namely, exponential penalty function method and primal dual interior point method. These two algorithms can accurately evaluate the flatness error of surfaces with complex geometry. However, when there are too many surface points, the performance will decline sharply. Samuel et al. (1999) developed an algorithm on the basis of computational geometry technology, which can be used for the minimum zone and function-oriented evaluation of straightness and flatness. However, it mainly focuses on the data measured by shape measuring instruments and CMM. Cui et al. (2002) applied the genetic algorithm on the basis of real number coding to the evaluation of flatness error. According to the mathematical definition of dimension and tolerance, the mathematical model of flatness error evaluation that fully meets the minimum zone condition is established, and the fitness function of genetic algorithm is given. Sun et al. (2020) applied genetic algorithm to structural optimization and further analyzed the genetic algorithm. However, it has not been tested on the actual surface topography data. Weber et al. (2002) introduced a unified linear approximation technique to evaluate the forms of straightness, flatness, roundness, and cylindricity. The nonlinear equation is linearized by Taylor expansion method and then solved. Experiments show that the result is equal to or better than the least squares method. According to the characteristics of flatness error evaluation, Wen et al. (2007) proposed an algorithm applying evolutionary strategy to flatness error evaluation. The algorithm is based on real number coding, adopts selection strategy and Gaussian mutation operator, and can evaluate the flatness error according to the objective function. Yue and Wu (2008) proposed an incremental algorithm for rapid flatness error evaluation, especially for the case of few measurement points. Cui et al. (2013) proposed an error evaluation method on the basis of PSO, verified the algorithm with experimental data and compared it with some typical optimization algorithms. The results show that the algorithm is better than other algorithms and easy to implement. Luo et al. (2012) proposed to apply the improved artificial bee colony algorithm to the evaluation of the minimum zone of flatness error. Experiments show that it is suitable for form and position error measuring instruments and CMM.

However, if the point cloud data are large and there is noise interference, then data preprocessing is very important. Chen et al. (2021) proposed a random forest algorithm, which can identify and classify data. Compared with the neural network algorithm, it is proved that the algorithm has high recognition accuracy. Gan et al. (2006) proposed a hierarchical clustering method on the basis of data field. In this method, the interaction between material particles and its field description method is introduced into the abstract number domain space, and the self-organizing hierarchical aggregation of data objects is realized by simulating the interaction and motion of objects in the virtual data field. It can well realize the classification of data.

In addition to the solution algorithms of the two standard methods, the wave front sensing method can also be used to evaluate the flatness error of micro/nano surface. Nutsch et al. (2007) and Zhang et al. (2021) improved the wave front sensing method proposed by Makyoh and Shack Hartmann and applied it to the flatness error evaluation of wafer surface.

To sum up, for the three-dimensional point cloud data of micro/nano surface, the corresponding segmentation algorithm is used for preprocessing according to the complexity, and then, the appropriate flatness error evaluation method is used for evaluation. On the basis of this, this paper uses PSO algorithm combined with minimum zone method to evaluate the flatness error. At the same time, considering that the actual measured micro/nano surfaces topography data are large and contains some noise, hierarchical clustering algorithm is used to improve PSO algorithm to meet the actual evaluation requirements.

## Data Preprocessing

**FIGURE 2:**
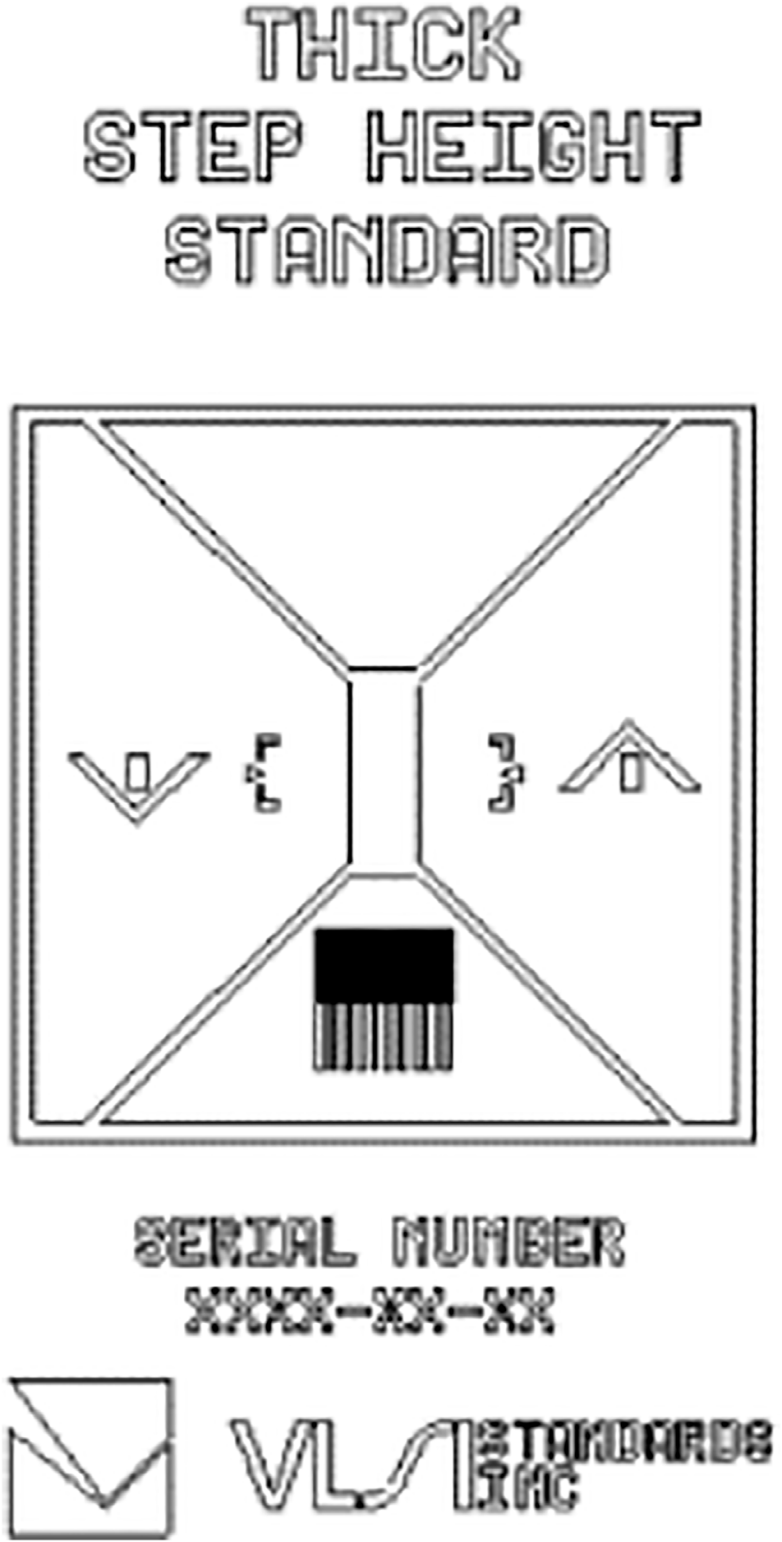
Schematic diagram of VLSI step height standard.

The standard sample used of this paper is SHS-1.8.0QC SHS of VLSI company. As shown in the Figure 2, the sample is mainly composed of accurate etching step height, monitoring instrument probe measurement dynamic and integrity test module. The nominal height is 1.8 μm (the calibration value is 1.767 ± 0.010 μm), the sample size is 25 mm, and SHS sample in the calibration zone is 2.5 mm long and 1 mm wide.

**FIGURE 3:**
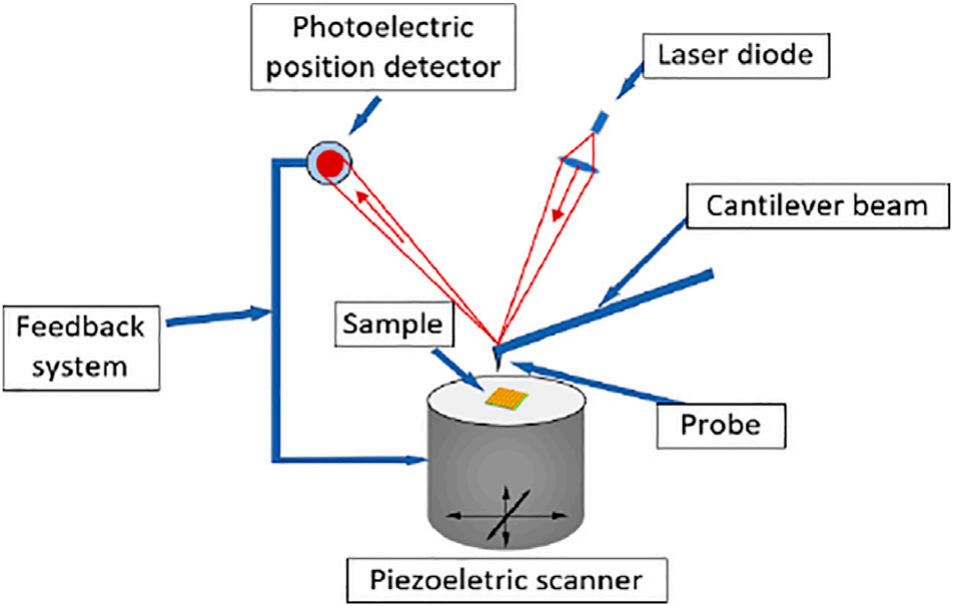
Working principal diagram of atomic force microscope.

Figure 3 shows the measurement principle of atomic force microscope (Shu et al., 2021). In the nano scale measurement range, the form of force between the two atoms will change with the change of distance. When the distance is long, the attraction plays a major role. At this time, the two atoms are in a state of mutual attraction. As the distance between the two atoms decreases, the attraction will gradually weaken. When the distance decreases to a certain value, the force between the atoms will decrease to zero. As the distance between the atoms continues to decrease, the force between the two atoms will show as repulsion force. AFM uses an elastic microcantilever that is very sensitive to micro force to feel the force between the probe tip atom and the sample surface atom. In the system, one end of the microcantilever is fixed, and the other end is equipped with a nano needle tip. The microcantilever will deflect when subjected to force. The micro topography of the sample surface can be measured by detecting the deflection of the microcantilever. However, the equipment used is a park NX10 atomic force microscope with a measurement range of 45 × 45 μm. The step width of the SHS sample exceeds the imaging range of atomic force microscope, so it is necessary to measure the edge of the SHS sample in forward and reverse directions respectively.

**FIGURE 4:**
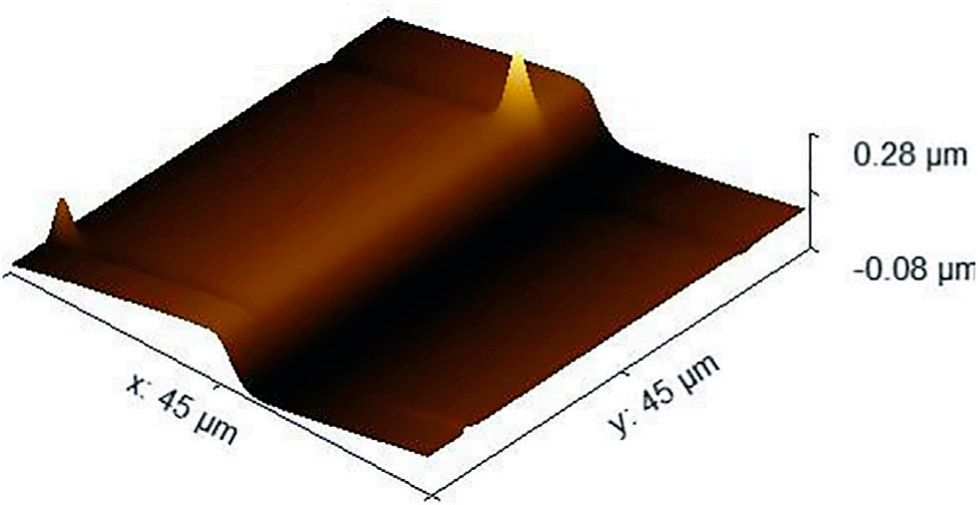
Atomic force microscope original image.

The scanning image data obtained by AFM cannot be directly used to evaluate the surface flatness error. Because of the influence of equipment noise and the instability of atomic force microscope measurement, some scratches or protrusions may appear in the scanned image in Figure 4. If we directly incorporate the problematic data into the evaluation method, then it will have a great impact on the accuracy of the evaluation results. Therefore, we need to preprocess the images obtained by AFM. At the same time, to reduce the contact damage to the probe and the sample of the measurement process, tapping mode is used for scanning.

**FIGURE 5:**
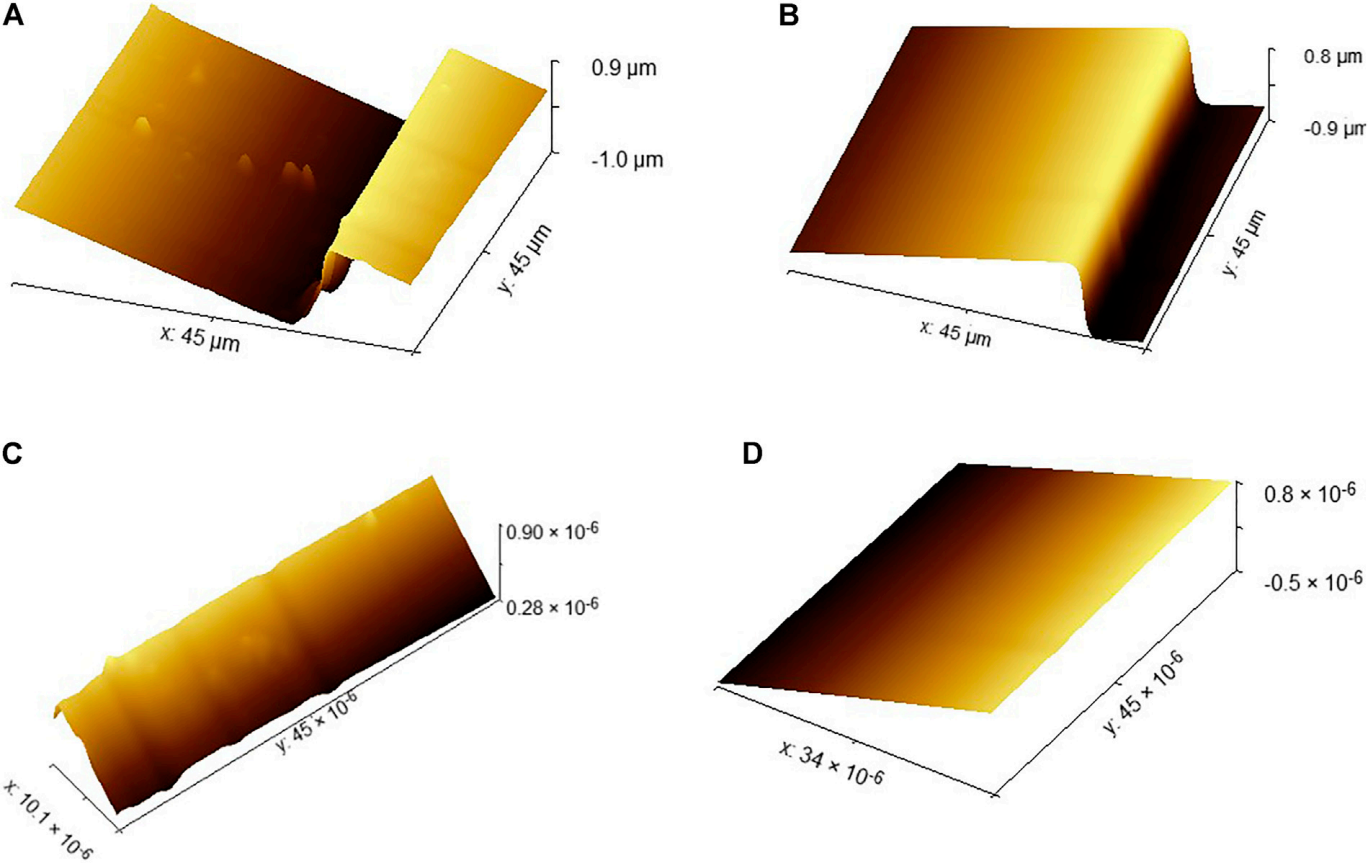
Separation process of required step surface topography data. **(A)** Left SHS sample surface. **(B)** Right SHS sample surface. **(C)** Left step surface. **(D)** Right step surface.

First, as shown in Figure 5, we separate the step surface data into the scanned images of the SHS sample surface on the left and right. In this way, the surface topography data on SHS samples can be separated to obtain the required step surface topography data, which is the maximum area required for flatness error evaluation.

Then, we consider the influence of sampling zone on the measurement results. For CMM flatness error measurement, many scholars have done similar research on sampling zone. Jalid et al. (2015) considered the influence of CMM sample size of flatness error. However, whether the micro/nano surface is the same or not needs to be proved by some experiments.

To analyze the influence of point cloud area size on flatness evaluation results of different micro nano surface topography data. This paper intends to classify the measured micro and nano surface topography datasets according to the region size. The total data point cloud area obtained from the measurement is 45 × 45 μm. According to the actual situation of the data point cloud, it is divided into 5 × 5 μm, 10 × 10 μm, and total SHS samples. The fifth part will carry out algorithm experiments on these three different sample sizes and analyze the influence of different sample sizes on the measurement results.

**FIGURE 6:**
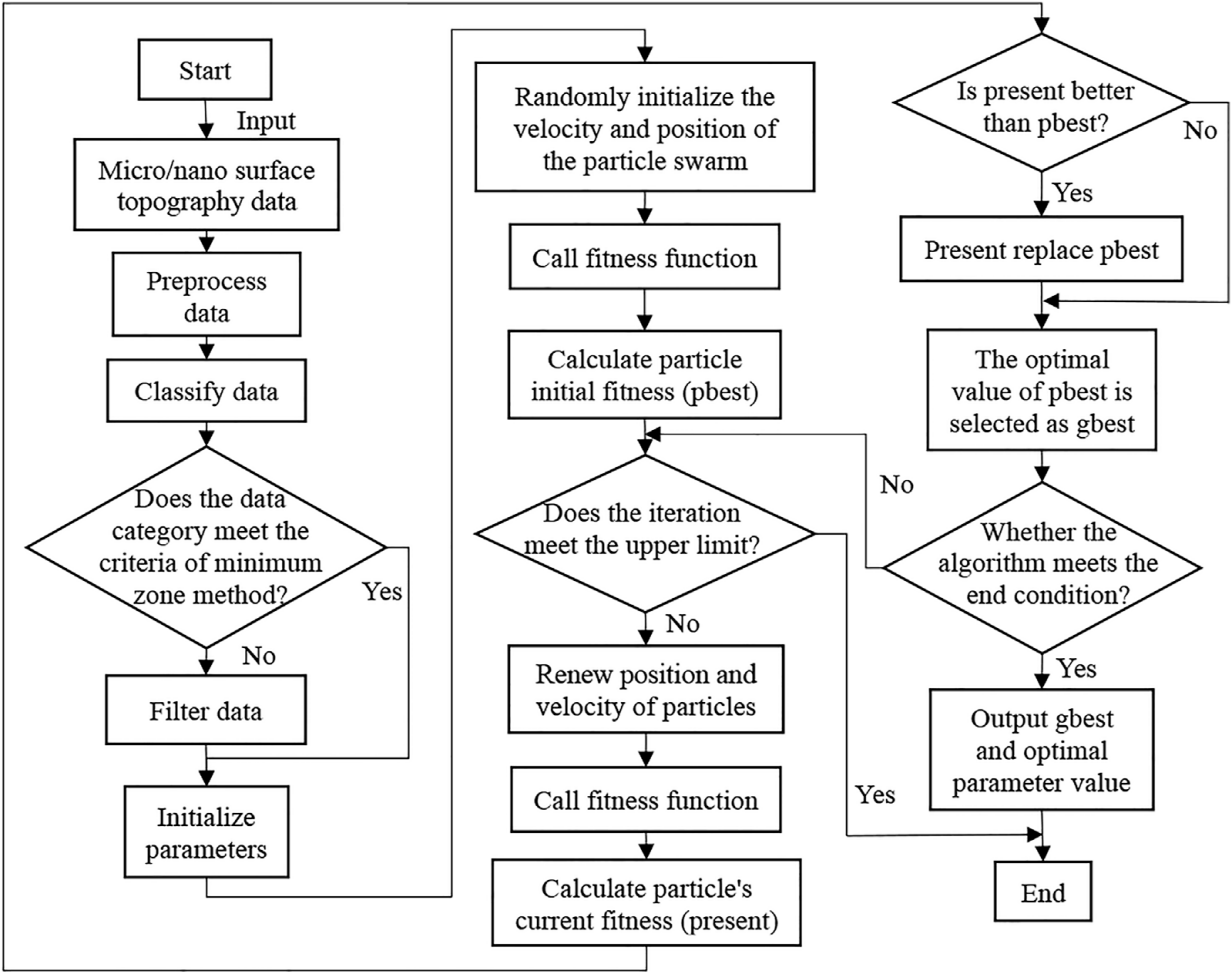
The flow chart of the minimum zone method based on improved particle swarm optimization algorithm total framework.

The flow chart of the total algorithm framework is shown in Figure 6. The principle of the algorithm is introduced in detail in *Flatness Error Evaluation Method*.

## Flatness Error Evaluation Method

### Minimum Zone Method

The traditional minimum zone method is to evaluate the sampling points of the surface measured by CMM equipment (Janani et al., 2019). The estimate of flatness error depends on the number and location of selected points in the part. The selection of sampling strategy is decided by the operator, and the size and location of sampling points affects the flatness error and uncertainty of the estimation.

**FIGURE 7:**
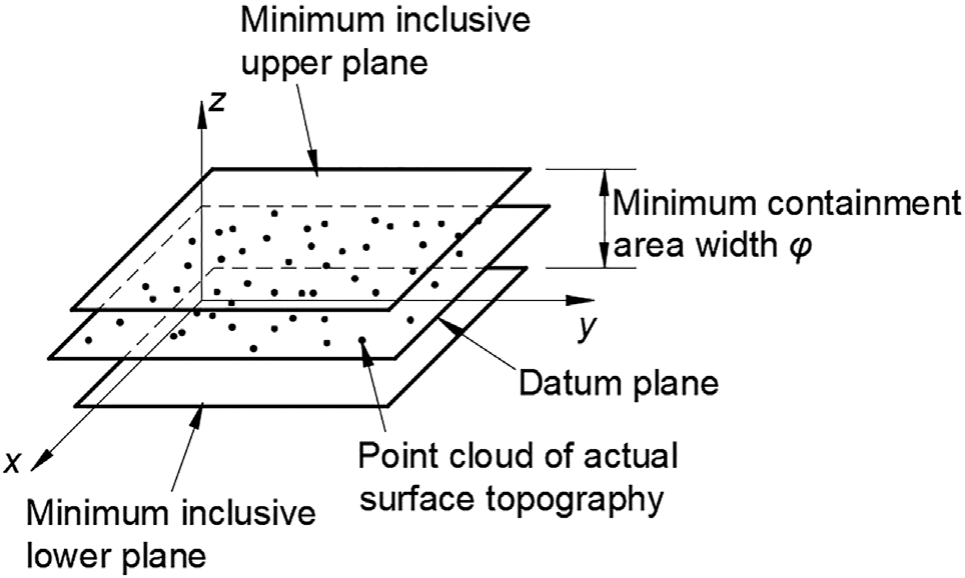
Minimum zone of flatness error.

According to the ISO standard, the minimum zone method is used to solve the flatness error. As shown in Figure 7, two parallel planes are determined on the upper and lower sides of the actual measured plane by a certain method, and all the measured points are contained, and the distance *d* between the two planes is the minimum, which is the calculated flatness error value.

However, the upper and lower parallel planes must have three or more points in contact with the actual measured plane and meet one of the following three criteria at the same time.

**FIGURE 8:**
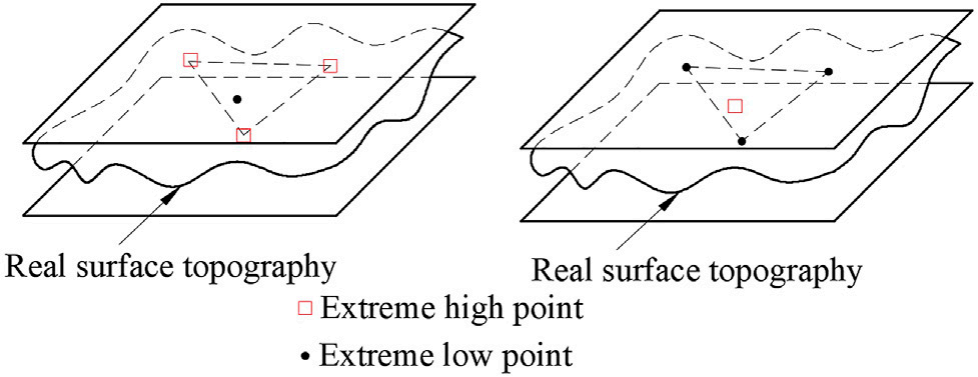
Triangular criteria.

**FIGURE 9:**
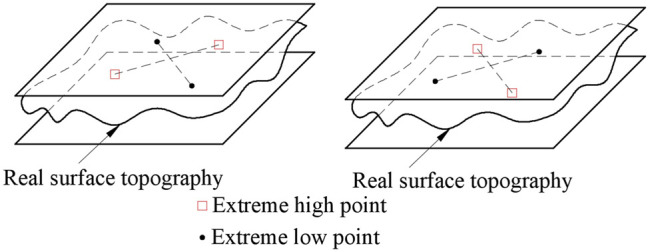
Cross criteria.

**FIGURE 10:**
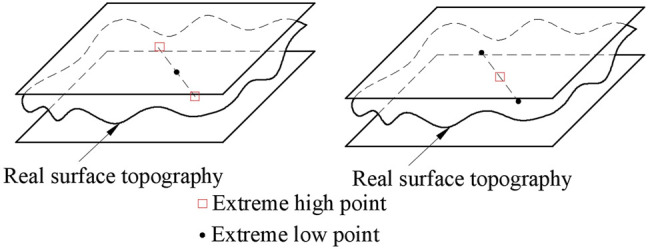
Linear criteria.

1) Triangle criteria: As shown in Figure 8, find out four points in the measured plane, which are three extreme high points and one extreme low point, or three extreme low points and one extreme high point. The projection points of the plane formed by three extreme low points or one extreme high point or one extreme low point corresponding to three extreme high points is just on the inner side or edge of the triangle composed of three extreme low points or three extreme high points.
2) Intersection criteria: As shown in Figure 9, find four points in the measured plane, which are two extreme high points and two extreme low points. The line of the two extreme high points and the line of the two extreme low points should be in the intersection state.
3) Straight line criteria: As shown in Figure 10, find three points in the measured plane, which are two extreme high points and one extreme low point, or two extreme low points and one extreme high point. It is required that in the *x-y* plane, one extreme low point can just be on the line of two extreme high points, or one extreme high point can just be on the line of two extreme low points.

### Particle Swarm Optimization Algorithm

PSO algorithm is a swarm-based stochastic optimization technology proposed by Eberhart (1995) and Kennedy (1995). PSO algorithm is a biologically inspired algorithm, which is solved by simulating the social behavior of animals, including insects, cattle, birds, and fish. These swarms will form a cooperative way to find food. Each member of the swarms will constantly change the search mode according to the learning experience of its own and other members.

We can design a massless particle to simulate the individual in the swarm. The particle has only two attributes: speed and position. Speed is the speed of movement and position is the direction of movement. Each particle separately searches for the optimal solution in the search space, records it as the current individual extreme value, shares the individual extreme value with other particles in the whole particle swarm, and finds the optimal individual extreme value as the current global optimal solution of the whole particle swarm. All particles in the particle swarm adjust their speed and position according to the current individual extreme value found by themselves and the current global optimal solution shared by the whole particle swarm.

First, the population *i* (*i* = 1, 2, 3, …, *n*) is initialized, and then, the optimal solution is found by iteration. In each iteration, the particle updates its position by tracking the two extreme values *pbest* and *gbest*. Among them, *pbest* is the best position that the particle has reached, and *gbest* is the best position for all particles in the whole swarm.

The specific particle velocity formula is as follows:

$$v_{i}=v_{i}+c_{1}\times \mathrm{rand}\left(\mathrm{pbes}t_{i}-x_{i}\right)+c_{2}\times \mathrm{rand}\left(\mathrm{gbes}t_{i}-x_{i}\right)$$ (1)

$$x_{i}=x_{i}+v_{i}$$ (2)

where *i* is the total number of particles in the swarm; $v_{i}$ is the velocity of particles; *rand* stands for generating a random number between (0,1); $x_{i}$ is the current position of particles; and $c_{1}$ and $c_{2}$ is the learning factors of swarm.

Equation 1 contains the following: memory term, which is the size and direction of the last velocity of the particle; self-cognition term, which is a vector from the current point to the best point of the particle itself, indicates that the action of the particle comes from its own experience; and swarm cognitive term, a vector from the current point to the best point of the population, which reflects the cooperation and knowledge sharing among particles. Particles determine their next movement through the best experience of themselves and their companions. This is the standard form of PSO algorithm.

### Minimum Zone Method Based on Improved Particle Swarm Optimization Algorithm

The surface data onto the step sample obtained by AFM is three-dimensional point cloud of large data points, which will appear local sharp bulges due to the influence of measurement process, surface dust, and other factors. If the PSO is used directly to evaluate, then the computation time and accuracy of the final algorithm will be affected by the computation time being too long or not convergent. To solve the above problems, the hierarchical clustering method is used to improve the PSO algorithm.

Hierarchical clustering uses Euclidean distance to calculate the distance between different types of data points. The data points in the minimum distance are combined. The calculation formula for Euclidean distance *d* is as follows:

$$d=\sqrt{{\left(x_{i}-x_{j}\right)}^{2}+{\left(y_{i}-y_{j}\right)}^{2}+{\left(z_{i}-z_{j}\right)}^{2}}$$ (3)

where *i* and *j* are two different points in the dataset; and *x*, *y*, and *z* are the coordinate values of the point cloud in the coordinate system.

To improve the effect of data points clustering, all data points $p_{i}=\left(x_{i},y_{i},z_{i}\right)$ (*i* = 1, 2, 3, …, *n*) can be hierarchical clustered according to the type of parallel plane.

**FIGURE 11:**
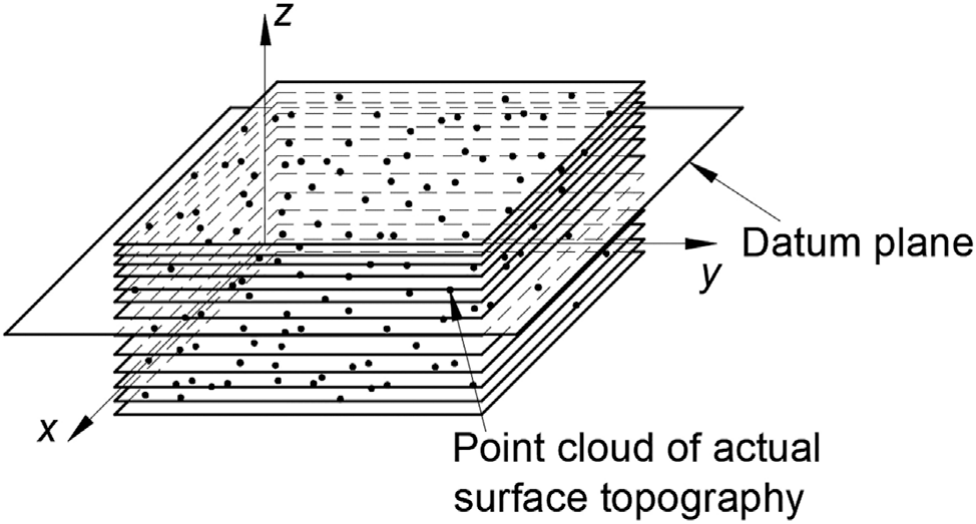
Schematic diagram of hierarchical clustering.

First, each data point is divided into an independent class $C\left(x_{i},y_{i},z_{i}\right)$ (*i* = 1, 2, 3, …, *n*). The total number of classes is equal to the number of point cloud points in the sampling zone, and then, the data points in the same plane are clustered on the basis of this. As shown in the Figure 11, the corresponding parallel plane classes are formed according to the reference plane.

Second, according to the triangle criterion of the minimum zone method, the existing classes with three or more data points on the same parallel plane are clustered into new classes *B* (*x*_*j*_, *y*_*j*_, *z*_*j*_) (*j* = 1, 2, 3, …, *m*), and the very high plane and the very low plane in the new class relative to the datum plane are the containment planes of the minimum zone method. The plane equation is as follows:

$$\{\begin{array}{c} z=a_{\max}x+b_{\max}y+c_{\max}\\ z=a_{\min}x+b_{\min}y+c_{\min} \end{array}$$ (4)

**FIGURE 12:**
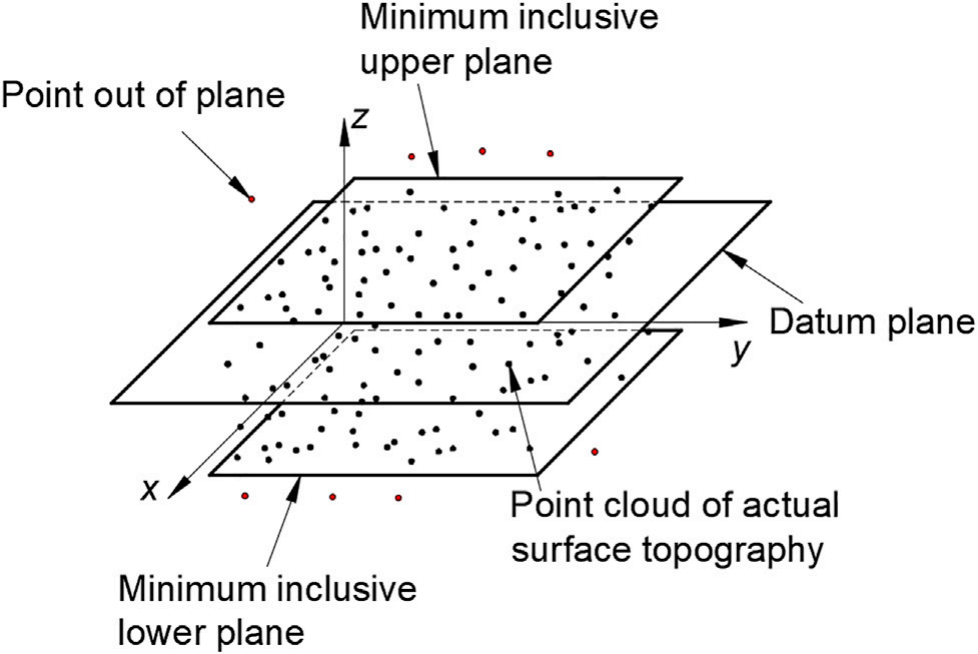
Filtering principle of surface topography point cloud data.

Third, as shown in the Figure 12, the points above and below the two planes according to the extremely high plane and the extremely low plane are filtered. The remaining points will be used as data points to evaluate the flatness error of the target. Next, we use PSO algorithm to evaluate the flatness error of the filtered data points.

The core of using PSO algorithm to evaluate flatness error is to find the corresponding evaluation function. The principle of the minimum zone method is to find two parallel planes in the spatial coordinate system (*x*, *y*, *z*). All points $p_{i}=\left(x_{i},y_{i},z_{i}\right)$ (*i* = 1, 2, 3, …, *n*) of the surface point cloud of the micro/nano surface are required to be contained in two parallel planes, and the distance φ between the two parallel planes is minimum. Then, the minimum distance φ is the flatness error value.

Therefore, the evaluation function of PSO can be defined as the distance between two parallel planes. Assuming that the reference plane *S* determined by the improved PSO algorithm is the position of the initial particle swarm, the equation is as follows:

$$z=a_{j}x+b_{j}y+c_{j}\left(j=1,2,...,n\right)$$ (5)

where *a*_*j*_, *b*_*j*_, and *c*_*j*_ are the coefficients of the corresponding plane equation.

The position of the plane is judged by all the points $p_{i}=\left(x_{i},y_{i},z_{i}\right)$ (*i* = 1, 2, 3, …, *n*) of the point cloud, that is, whether the plane is in the point cloud or outside the point cloud. Then, two parallel planes are determined from the plane, and the condition that all points of the point cloud are just included between the two parallel planes is met at the same time. The equations of two parallel planes are as follows:

$$\{\begin{array}{c} z=a_{j}x+b_{j}y+c_{j1}\\ z=a_{j}x+b_{j}y+c_{j2} \end{array}\left(j=1,2,...,n\right)$$ (6)

The distance between two parallel planes is as follows:

$$D=\left|c_{j1}-c_{j2}\right|\left(j=1,2,...,n\right)$$ (7)

The fitness function is the minimum of two parallel planes, that is, the flatness error of the final solution:

$$D_{\min}=\min\left|c_{j1}-c_{j2}\right|\left(j=1,2,...,n\right)$$ (8)

When we know the initial particle swarm location and the corresponding fitness function, we can use the PSO algorithm to find the optimal solution.

## Experimental Results and Analysis

### Evaluation Results and Analysis of Flatness Error

**FIGURE 13:**
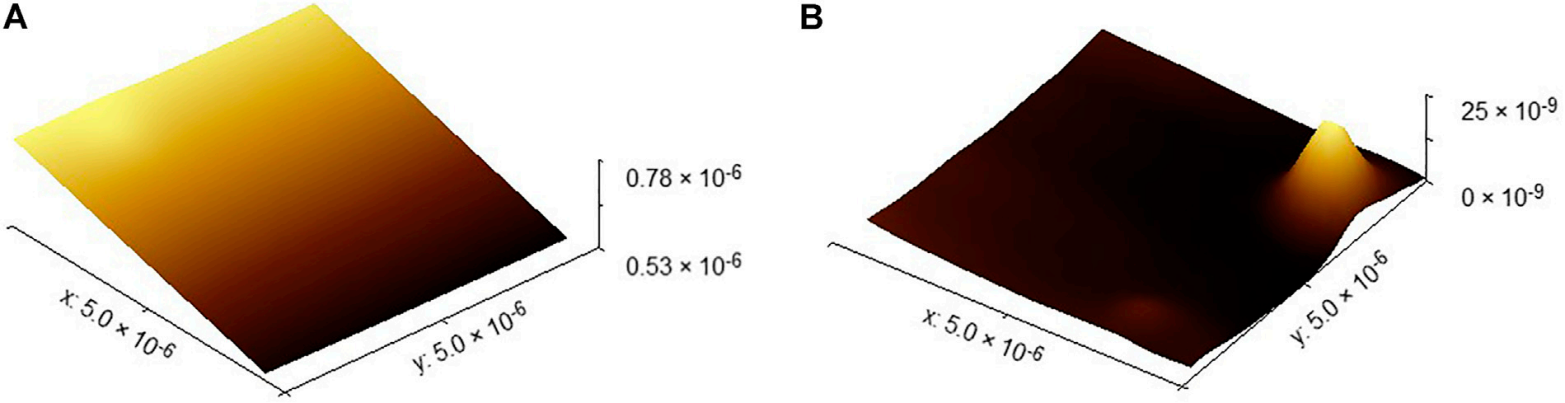
Surface topography of the SHS sample of 5 × 5 μm. **(A)** Original surface topography of the SHS sample of 5 × 5 μm. **(B)** Surface topography of the SHS sample of 5 × 5 μm after leveled.

First, the surface topography data onto the SHS sample is segmented into a surface topography data of 5 × 5 μm. As shown in Figure 13A, the plane of the surface topography data obtained directly is not parallel to the *x-y* plane, so it is not convenient to evaluate the flatness error. The plane level algorithm is used to smooth the surface. It is computed from all the image points and is subtracted from the data. In this way, the processed surface data will be parallel to the *x-y* plane. At the same time, Figure 13B shows that, for the convenience of evaluation, fix zero and zero mean value algorithm is used to translate all the surface topography data to the *x-y* plane. The principle of the algorithm just added a constant to all the data to move the minimum value of zero, respectively.

Second, improve the parameters of PSO algorithm: to improve the speed of the algorithm and hope that the result is as close to the ideal optimal value as possible, the initial range of particles is determined by the least squares method. Set the initial particle population *n* = 30 and the dimension of particle search space to 3. The number of final iterations is set to 50. The value range of inertia weight is 0.4–0.9.

**FIGURE 14:**
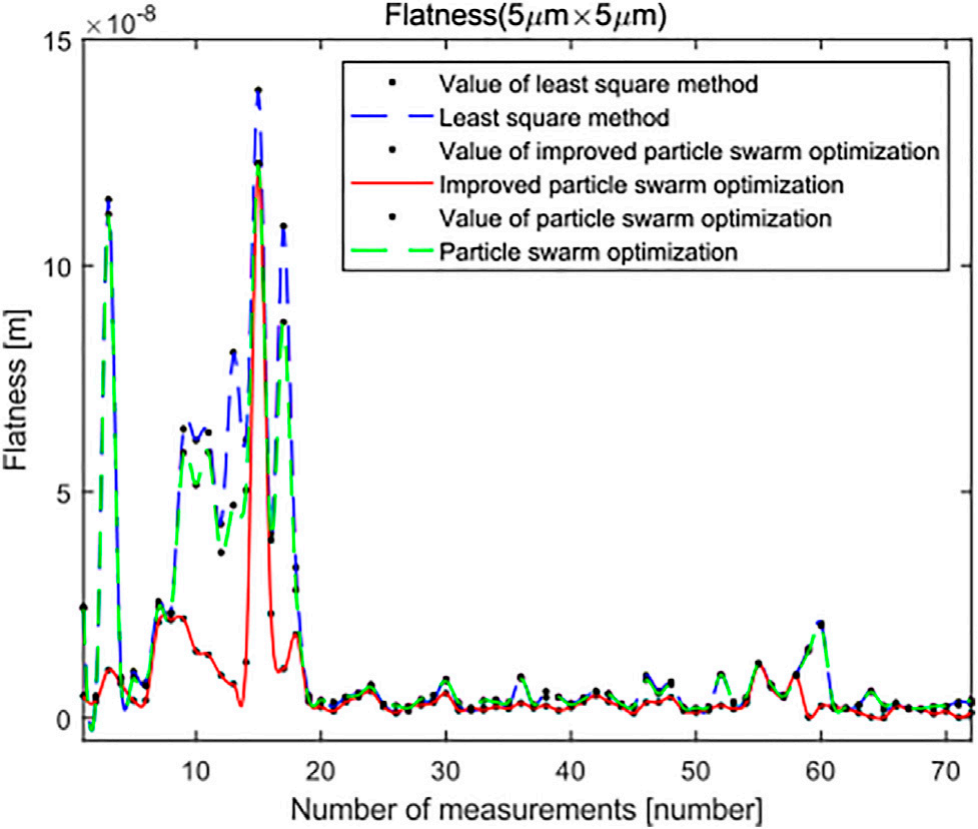
Flatness error evaluation of the SHS sample of 5 × 5 μm.

**FIGURE 15:**
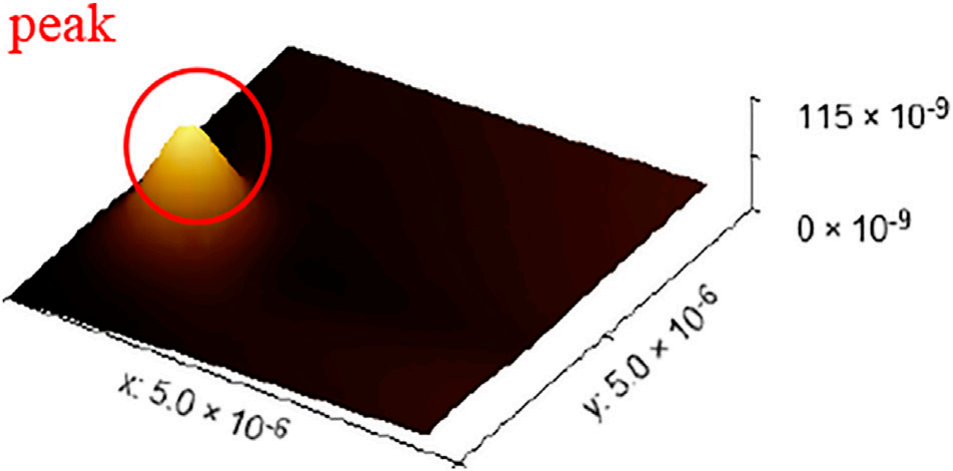
Reasons for the impact of measurement results.

Last, the surface topography data of 72 groups of leveled SHS samples of 5 × 5 μm were imported by standard least squares method, standard PSO algorithm, and minimum zone method based on the improved PSO algorithm. The flatness error evaluation results are shown in Figure 14. The evaluation results of standard PSO algorithm and standard least squares method are roughly the same, and the results based on the improved PSO algorithm are obviously better than the other two methods. However, in the 15th group of data in the figure, the results based on the improved PSO algorithm have also changed greatly. The specific reasons for this are shown in Figure 15. The surface topography of SHS leveled samples of 5 × 5 μm consists of 784 data points. Because of the influence of dust or noise on the surface topography of SHS leveled samples, a peak will be formed in the point cloud. Compared with the other two algorithms, the minimum zone method based on improved PSO combines hierarchical clustering and minimum region method, which can preliminarily remove the impact of noise on the results and has better robustness. However, because the number of surface data points is too small, it will have a great impact on the results when evaluating the surface shape area, so this group of results will fluctuate to a certain extent. However, its fluctuation range is still smaller than that of standard least squares method and standard PSO algorithm.

**FIGURE 16:**
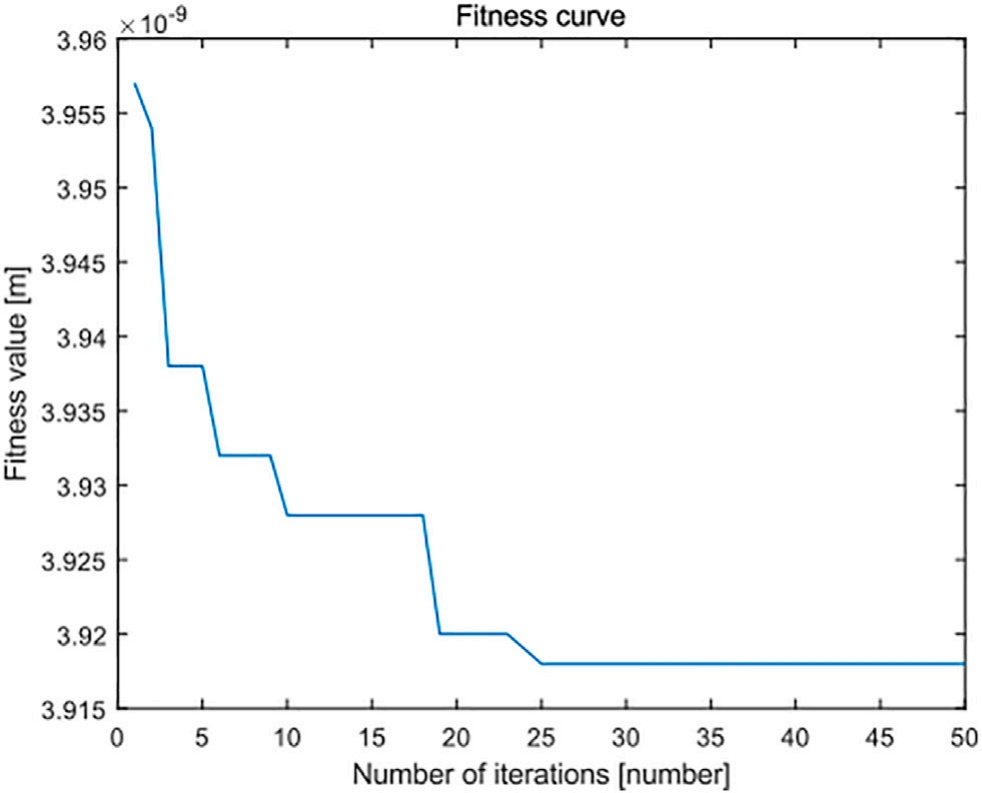
Fitness error curve of improved particle swarm optimization algorithm in flatness error evaluation of the SHS sample of 5 × 5 μm.

**FIGURE 17:**
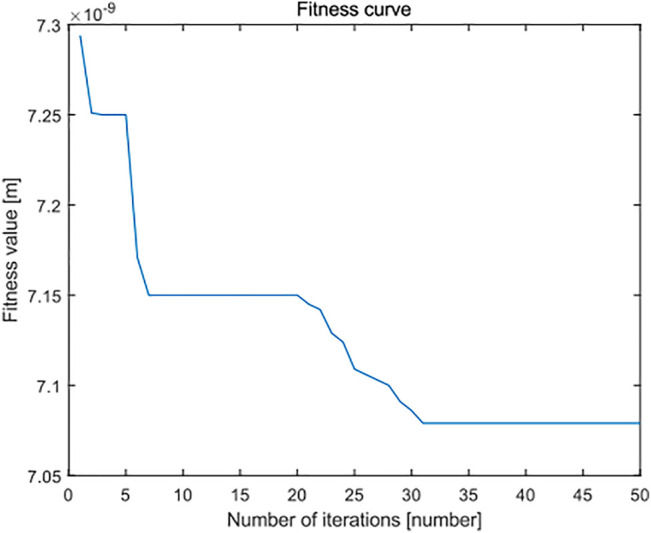
Fitness error curve of standard particle swarm optimization algorithm in flatness error evaluation of the SHS sample of 5 × 5 μm.

In the overall view of the curve, the minimum zone method based on the improved PSO algorithm is smaller than the standard least squares method and standard PSO algorithm in the flatness error evaluation results of leveled SHS sample surface topography of 5 × 5 μm, and most of the evaluation results are very stable and robust. It can see from Figure 16 and Figure 17 that, when the number of iterations is close to 25, the minimum region method of the improved PSO algorithm has fully converged, whereas the corresponding standard PSO algorithm can reach convergence only after about 30 times, and the convergence result is significantly worse than the minimum region method based on the improved PSO algorithm. This shows that the algorithm can realize the optimal calculation of flatness error.

**FIGURE 18:**
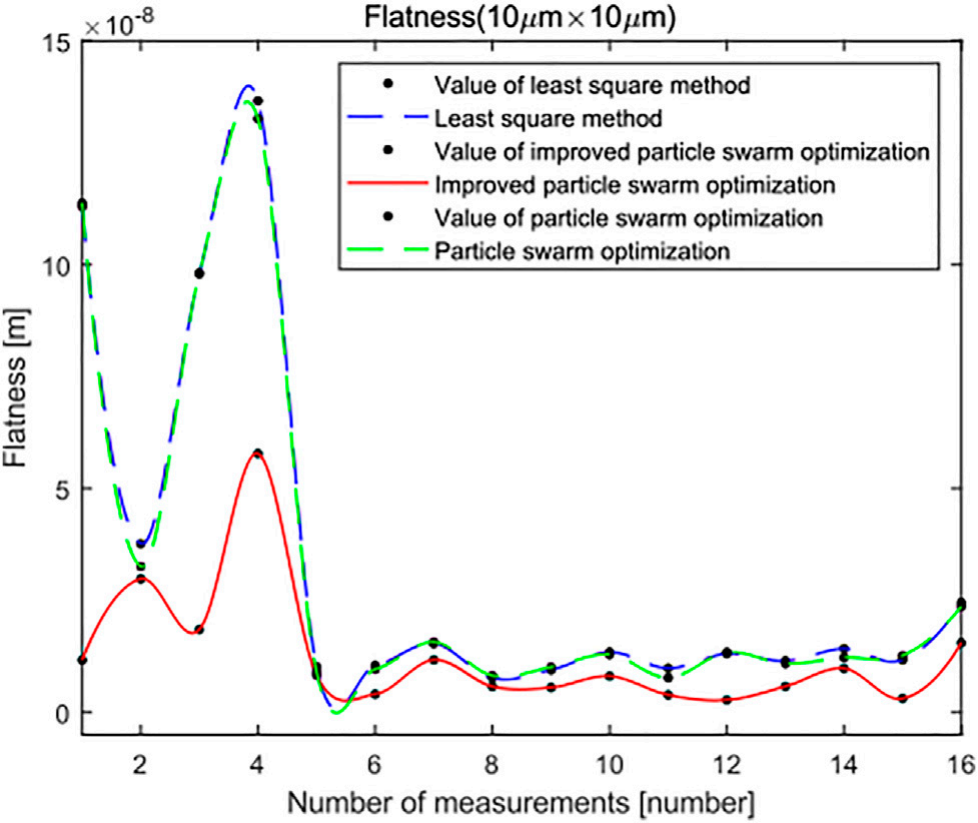
Flatness error evaluation of the SHS sample of 10 × 10 μm.

**FIGURE 19:**
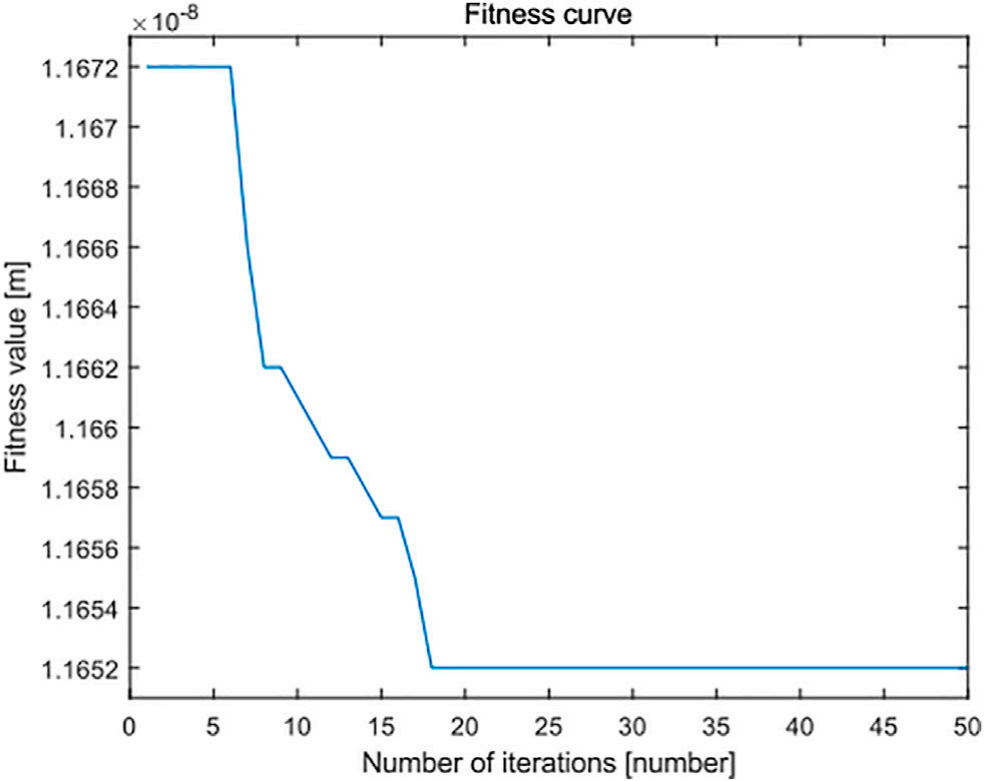
Fitness curve of improved particle swarm optimization algorithm in flatness error evaluation of the SHS sample of 10 × 10 μm.

In the same way, the surface topography data of 16 groups of leveled SHS samples of 10 × 10 μm are imported into the standard least squares method, the standard PSO algorithm and the minimum zone method based on the improved PSO algorithm. The flatness error evaluation results are shown in Figure 18. There are 3,249 points in the surface topography data of leveled SHS samples of 10 × 10 μm. In the flatness error evaluation results of leveled SHS sample surface topography of 10 × 10 μm, because there are many points, the flatness error evaluated by the minimum zone method based on the improved PSO algorithm is significantly smaller than that evaluated by the standard least squares method and the standard PSO algorithm and has good robustness. At the same time, Figure 19 shows the flatness error evaluation results of leveled SHS samples of 10 × 10 μm based on the improved PSO algorithm also converge rapidly and completely, and the optimal calculation of error can be realized.

For the total surface topography data on leveled SHS samples, standard least squares method, standard PSO algorithm, and least squares method on the basis of the improved PSO algorithm are introduced, respectively. The flatness error evaluation results are shown in Table 1.

**TABLE 1:** The three algorithms are based on the flatness error evaluation results of the maximum SHS sample surface topography data.

Data size	Least squares method (nm)	Minimum zone method based on particle swarm optimization algorithm (nm)	Minimum zone method based on improved particle swarm optimization algorithm (nm)
10 × 45 μm	1100.0	249.60	156.60
35 × 45 μm	172.50	118.20	105.00
Total size	378.61	147.40	116.47

**FIGURE 20:**
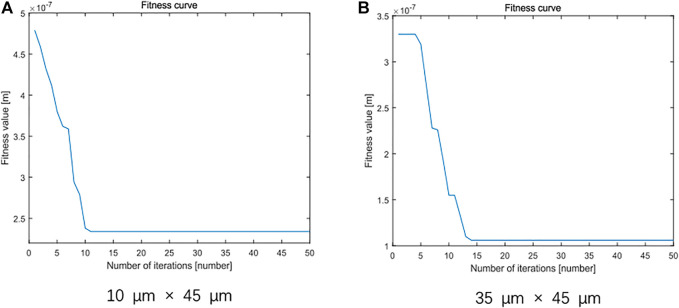
Comparison of flatness error for SHS sample. **(A)** 10 × 45 μm. **(B)** 35 × 45 μm.

The flatness error evaluation result of the minimum zone method based on the improved PSO algorithm is less than that of the standard least squares method and the standard PSO algorithm. Moreover, it can be seen from Figure 20 that the flatness error evaluation result of the minimum zone method based on the improved PSO algorithm can converge quickly and completely, and the optimal calculation of flatness error is realized.

The average value of flatness error evaluation result in the surface data onto leveled SHS samples of 5 × 5 μm and 10 × 10 μm is shown in Table 2.

**TABLE 2:** The average value of flatness error evaluation results of three algorithms under different SHS sample surface area.

Data size	Least squares method (nm)	Minimum zone method based on particle swarm optimization algorithm (nm)	Minimum zone method based on improved particle swarm optimization algorithm (nm)
5 × 5 μm (784 points)	16.320	14.700	6.7398
10 × 10 μm (3249 points)	33.542	32.700	12.620
45 × 45 μm (63,232 points)	378.61	147.40	116.47

Through the above experimental results, it can be observed that the size of leveled SHS sample data will directly affect the final evaluation results. As shown in Figure 14, it will even cause great changes to the measurement results of a certain time. In addition, with the increase of micro/nano surface topography data, the flatness error evaluation results of the three methods are also increasing. It is still uncertain which area size is the best. However, with the increasing area, the flatness error evaluation result based on the improved PSO algorithm will be more accurate.

### Uncertainty Analysis of Measurement Results

Considering that the surface topography of leveled SHS samples has been evaluated for many times, the repeatability of flatness error evaluation results has been evaluated. Therefore, class A standard uncertainty can be used for evaluation. The basic method of class a standard uncertainty evaluation is to calculate the experimental standard deviation and average experimental standard deviation of single measurement results. Bessel formula is used to calculate the estimated standard deviation of a single measurement:

$$s\left(x\right)=\sqrt{\frac{\sum_{i=1}^{n}{\left(x_{i}-\bar{x}\right)}^{2}}{n-1}}$$ (9)

where *n* is the number of measurements, $\bar{x}$ is the average of measurement results, and *x*_*i*_ is the results of each measurement.

Therefore, the standard uncertainty of a single measurement can be obtained from Eq. 10:

$$u\left(x\right)=s\left(x\right)=\sqrt{\frac{\sum_{i=1}^{n}{\left(x_{i}-\bar{x}\right)}^{2}}{n-1}}$$ (10)

In addition, the standard deviation of the mean is as follows:

$$u\left(\bar{x}\right)=s\left(\bar{x}\right)=s\left(x\right)/\sqrt{n}=\sqrt{\frac{\sum_{i=1}^{n}{\left(x_{i}-\bar{x}\right)}^{2}}{n\left(n-1\right)}}$$ (11)

If we can get the corresponding confidence probability, then we can get the expanded uncertainty from the estimated standard deviation of a single measurement.

U=ku(x) (12)

where *k* is the confidence factor; it depends on the confidence probability and the number of samples.

For the surface topography data of leveled SHS samples of 5 × 5 μm, the class A standard uncertainty values of the flatness error evaluation results of the three algorithms are shown in Table 3.

**TABLE 3:** Two algorithms for evaluating the uncertainty of flatness error of the SHS sample of 5 × 5 μm.

Uncertainty type	Least squares method (nm)	Minimum zone method based on particle swarm optimization algorithm (nm)	Minimum zone method based on improved particle swarm optimization algorithm (nm)
The standard uncertainty of a single measurement u(x)	28.047	24.139	14.879
The standard deviation of the mean $u\left(\bar{x}\right)$	3.3054	2.8448	1.7535
the expanded uncertainty U99 (99.9%, *k* = 3.496)	98.052	84.390	52.017

For the surface topography data of leveled SHS samples of 10 × 10 μm, the class A standard uncertainty values of the flatness error evaluation results of the three algorithms are shown in Table 4.

**TABLE 4:** Two algorithms for evaluating the uncertainty of flatness error of the SHS sample of 10 × 10 μm.

Uncertainty type	Least squares method (nm)	Minimum zone method based on particle swarm optimization algorithm (nm)	Minimum zone method based on improved particle swarm optimization algorithm (nm)
The standard uncertainty of a single measurement u(x)	42.079	41.708	13.927
The standard deviation of the mean $u\left(\bar{x}\right)$	10.520	10.427	3.4817
The expanded uncertainty U99 (99.9%, *k* = 3.496)	147.11	145.81	48.689

It can be seen from Tables 3 and 4 that, in the flatness error evaluation results of leveled SHS sample surface topography of 5 × 5 μm and 10 × 10 μm, the evaluation uncertainty of the minimum area method based on the improved PSO algorithm is far less than that of the standard least squares method and standard PSO algorithm. The experimental results show that the flatness error evaluation result of the minimum zone method based on the improved PSO algorithm is closer to the measured true value and has better reliability. At the same time, with the increasing area of surface topography, the reliability of flatness error evaluation results of the minimum zone method based on the improved PSO algorithm becomes better, whereas the standard least squares method and standard PSO algorithm become worse.

## Conclusion

To meet the needs of flatness error evaluation of micro/nano surface, a minimum zone method on the basis of the improved PSO algorithm is proposed to evaluate the flatness error of micro/nano surface. The main contributions of this paper are as follows.

On the basis of the analysis of various methods, combined with hierarchical clustering, minimum zone method, and PSO algorithm, a minimum zone method on the basis of the improved PSO algorithm is proposed to evaluate the flatness error of micro/nano surface.

The flatness error evaluation results of standard least squares method, standard PSO algorithm, and minimum zone method on the basis of the improved PSO algorithm are compared. The results show that the evaluation results of the minimum zone method based on the improved PSO algorithm is better than the standard least squares method and the standard PSO algorithm, and the robustness of the minimum zone method based on the improved PSO algorithm is better than the least squares method. From the uncertainty results, the flatness error evaluation result of the minimum zone method based on the improved PSO algorithm is closer to the measured real value than the other two methods.

The standard class A uncertainty, the corresponding extended uncertainty, and flatness error of the three algorithms under leveled SHS sample surface data of 5 × 5 μm and 10 × 10 μm are studied. The results show that the flatness error will increase with the increase of micro/nano surface topography data. The reliability of the evaluation results of the minimum zone method based on the improved PSO algorithm is increasing.

However, there are still some limitations in this paper, which need to be further discussed. In this paper, only the evaluation results of 5 × 5 μm, 10 × 10 μm, and total leveled SHS sample surface topography data are considered, and the optimal surface topography area size for flatness error evaluation is not determined. The filtering method is not considered to eliminate the influence of noise on the evaluation results. In future research, we will further explore the impact of different size surface topography regions on the evaluation results and develop appropriate filters to meet the needs of micro/nano surface topography evaluation ISO, 2017.

## Data Availability

The raw data supporting the conclusions of this article will be made available by the authors, without undue reservation.
